# Composition-Orientation Induced Mechanical Synergy in Nanoparticle Brushes with Grafted Gradient Copolymers

**DOI:** 10.1021/acs.macromol.3c01799

**Published:** 2023-11-29

**Authors:** Rongguan Yin, Yuqi Zhao, Jaepil Jeong, Jirameth Tarnsangpradit, Tong Liu, So Young An, Yue Zhai, Xiaolei Hu, Michael R. Bockstaller, Krzysztof Matyjaszewski

**Affiliations:** † Department of Chemistry, Carnegie Mellon University, 4400 Fifth Avenue, Pittsburgh, Pennsylvania 15213, United States; ‡ Department of Materials Science and Engineering, Carnegie Mellon University, 5000 Forbes Avenue, Pittsburgh, Pennsylvania 15213, United States

## Abstract

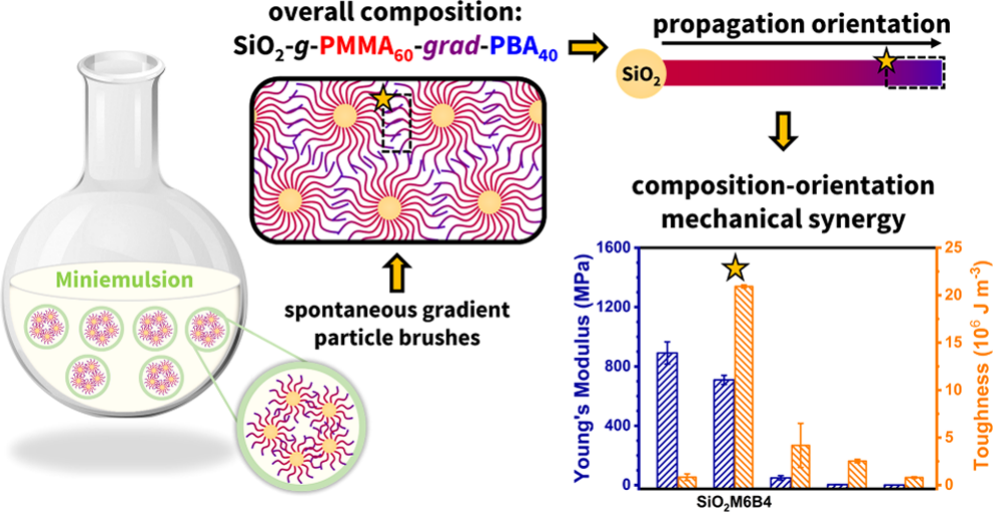

Gradient poly(methyl methacrylate/*n*-butyl acrylate) copolymers, P(MMA/BA), with various compositional ratios, were grafted from surface-modified silica nanoparticles (SiO_2_-*g*-PMMA-*grad*-PBA) via complete conversion surface-initiated activator regenerated by electron transfer (SI-ARGET) atom transfer radical polymerization (ATRP). Miniemulsion as the reaction medium effectively confined the interparticle brush coupling within micellar compartments, preventing macroscopic gelation and enabling complete conversion. Isolation of dispersed and gelled fractions revealed dispersed particle brushes to feature a higher Young’s modulus, toughness, and ultimate strain compared with those of the “gel” counterparts. Upon purification, brush nanoparticles from the dispersed phase formed uniform microstructures. Uniaxial tension testing revealed a “mechanical synergy” for copolymers with MMA/BA = 3:2 molar ratio to concurrently exhibit higher toughness and stiffness. When compared with linear analogues of similar composition, the brush nanoparticles with gradient copolymers had better mechanical properties, attributed to the synergistic effects of the combination of composition and propagation orientation, highlighting the significance of architectural design for tethered brush layers of such hybrid materials.

## Introduction

Nanoparticle brushes represent a class of hybrid materials that combine functional polymers with inorganic/organic nanostructured compounds.^1−5^ Over the decades, with the advancements in nanoparticle surface modification strategies^6−9^ and various surface-initiated polymerization methods,^10−14^ polymer nanohybrids with tailored architectures have gathered significant attention.^15^ One key advantage is that the nanoparticles (NPs) can retain their inherent properties while polymer grafts afford improved stability and processability of nanoparticle-based materials.^16−18^ Furthermore, the connectivity and interactions between polymer and particle components can generate synergistic effects and result in novel properties that render polymer-modified nanoparticles (also brush particles) interesting for applications such as lubricants, lithography, battery electrodes, and gas separation.^19−22^

**Scheme 1:**
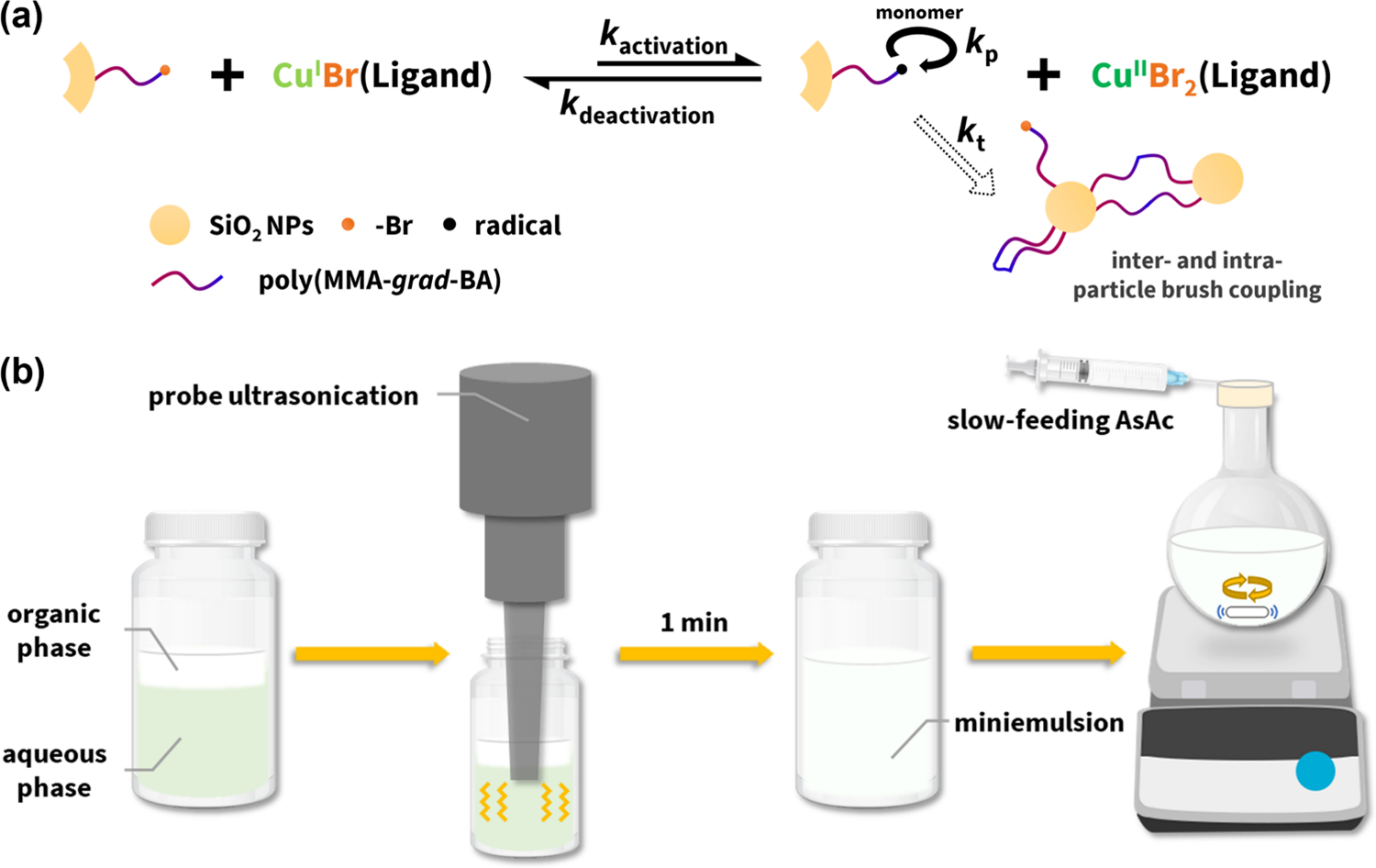
Synthesis of Spontaneous Gradient Copolymer-Grafted Particle Brushes SiO_2_-*g*-PMMA-*grad*-PBA (a) Mechanism of surface-initiated atom transfer radical polymerization (SI-ATRP) and (b) illustration of preparation of miniemulsion.

In terms of grafting (co)polymer chains from nanoparticle interfaces, surface-initiated atom transfer radical polymerization (SI-ATRP) stands out as the preferred method due to the formation of polymers with predetermined molecular weight, narrow molecular weight distribution, high fidelity of polymer chain ends, high grafting density, as well as its compatibility with a broad spectrum of functional groups and monomers.^23−28^ However, the termination process in SI-ATRP involving intra- and especially interparticle brush coupling can significantly increase the viscosity during polymerization (cf. Scheme 1a).^29^ This can result in macroscopic gelation if the polymerization is not quenched at a relatively low monomer conversion, typically below 20%. Consequently, this limitation presents a barrier to the scale-up of brush particle materials. Various strategies were demonstrated to achieve high monomer conversion in particle brush synthesis, including polymerization at high dilution, under high pressure, or incorporation of a small molecule “sacrificial” initiator.^30−32^ However, while these methods have advanced brush particle preparation, the cost and viability remain a challenge, especially for more complex systems such as gradient copolymer brush particles.

Gradient copolymers can be formed in a controlled/living system by either feeding one comonomer or spontaneously, as in the case of comonomers with very different reactivity ratios. The latter approach typically needs very high comonomer conversion.^33−35^ In reversible-deactivation radical polymerization (RDRP),^36^ comonomers with significantly different reactivity ratios are consumed at different rates.^37−39^ A comonomer that reacts faster is predominantly incorporated at the beginning of copolymer chains, while the other comonomer is consumed more slowly, resulting in a spontaneously generated gradient copolymer sequence structure. The gradual compositional change along the chain imparts intriguing physical properties, including broad glass transition, phase transition, and interfacial activity.^40−42^ These unique features make gradient copolymers suitable for applications, such as compatibilizers for immiscible polymer blends, stabilizers for emulsions/dispersions, thermoplastic elastomers, adhesives, and wetting agents.^34^ Recently, there has been a growing interest in self-healable poly(methyl methacrylate/*n*-butyl acrylate) [P(MMA/BA)] copolymers.^43−45^ Compared to the alternating and statistical copolymer architectures, the gradient P(MMA/BA) demonstrated superior mechanical performance attributed to the segregation of segments to form nanoscopic PMMA-rich cluster regions.^46,47^ Gradient P(MMA/BA) copolymers thus hold promise as a platform for self-healing materials due to the simple synthesis and superior mechanical properties.^48^ When applied to brush particle systems, P(MMA/BA) gradient copolymers have shown to endow hybrid materials with dual “self-heal” and “shape memory” ability, thus providing a path to multifunctional materials.^49^

In previous studies, the “gradient” architecture in copolymer particle brushes was attained by slowly feeding MMA monomers into polymerizing BA solutions.^50^ Despite the difference in reactivity ratios (defined as the ratio of self-propagation versus cross-propagation rate constant, *r*_MMA_ = 1.79 and *r*_BA_ = 0.30),^51^ the initial stage of copolymerization was dominated by PBA-rich segments close to the particle surface (i.e., SiO_2_-*g*-PBA-*grad*-PMMA) due to the higher concentration of BA monomers. This resulted in a unique “reverse gradient” architecture compared to the traditional “spontaneous gradient” architecture with PMMA-rich segments at the beginning of growing chains, when considering the propagation orientation in copolymerization.^52^ However, comonomer conversions were maintained below 20% to prevent gelation. Herein, to address this challenge and achieve spontaneous gradient copolymer-grafted particle brushes (i.e., SiO_2_-*g*-PMMA-*grad*-PBA) at complete comonomer conversions, the miniemulsion technique was employed for the specific case of surface-initiated activators regenerated by electron transfer (SI-ARGET) ATRP.^53−55^ The potential cross-coupling between particle brushes was confined within the physical boundaries of each miniemulsion droplet, thereby preventing the macroscopic gelation of the entire reaction mixture.^56^ The composition of the grafted brush layer for five different initial PMMA/PBA molar ratios revealed gradient sequences along the copolymer propagation orientation. Upon redispersing all of the reaction products in a suitable solvent, about 90 wt % of them formed stable solutions/dispersions, with a small fraction (∼10 wt %) of nanoparticles swollen even under high dilution and prolonged sonication. Subsequent analysis of both the dispersed and “gel” particle brush fraction was performed following separation by centrifugation. The purified dispersed particle brushes had predetermined copolymer compositions, narrow molecular weight distributions, and uniform size distributions that were devoid of aggregation. Characterization of the thermal properties revealed broad glass transitions that were attributed to the spontaneous gradient copolymer sequence structure. Particularly noteworthy was the remarkable mechanical synergy observed when the MMA/BA molar ratio was 60:40, indicating that the tailoring of the copolymer brush sequence could provide a path toward novel functional material systems.

## Results and Discussion

### Synthetic Procedures and Kinetic Studies

**Figure 1:**
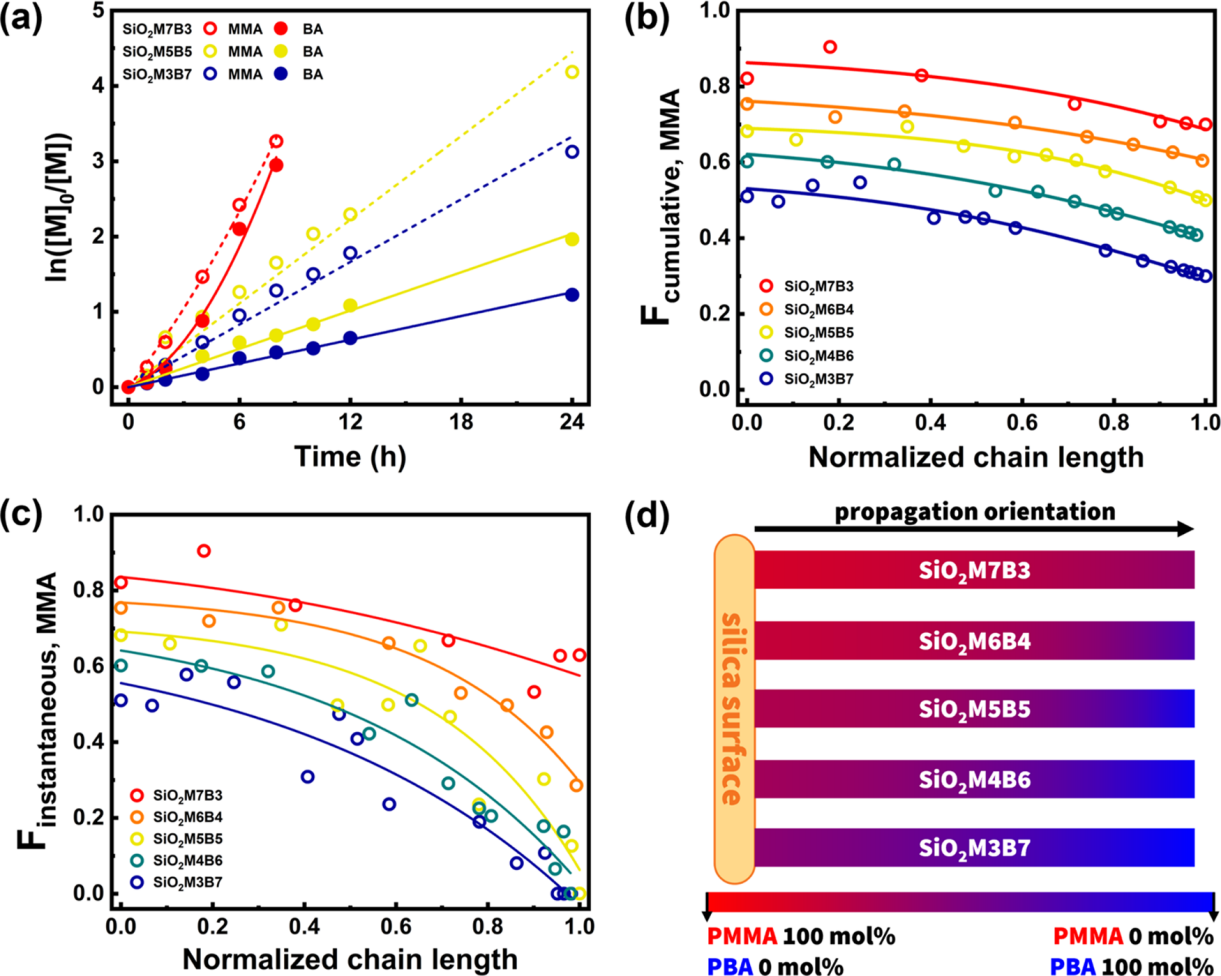
Spontaneous gradient poly(methyl methacrylate/*n*-butyl acrylate) copolymer-grafted silica nanoparticle brushes (SiO_2_-*g*-PMMA-*grad*-PBA) by full conversion miniemulsion SI-ATRP, with MMA/BA molar ratios of 70:30 (red), 60:40 (orange), 50/50 (yellow), 40:60 (green), and 30:70 (blue). Reaction conditions using [MMA]_0_/[BA]_0_ = 50:50 as an example: miniemulsion SI-ARGET ATRP, SiO_2_–Br 320 mg, BA 2.7 mL, MMA 2.0 mL, comonomers 18.7 vol % in H_2_O, [MMA]_0_/[BA]_0_/[SiO_2_–Br, ≈0.45 Br/nm^2^]_0_/[Cu^II^Br_2_(TPMA)]_0_ = 500:500:1:0.8, [HD]_0_ = 10 wt % and [SDS]_0_ = 6.2 wt % relative to comonomers, and [NaBr]_0_ = 0.1 M; ascorbic acid (AsAc) as a reducing agent was injected using a syringe pump (rate = 0.5 equiv h^–1^ compared with [Cu^II^Br_2_(TPMA)]_0_ for 10 h); the miniemulsion was prepared by ultrasonication; *T* = 50 °C. (a) Semilogarithmic kinetic plots within 24 h (complete kinetics in Figure S2). (b) Cumulative and (c) instantaneous incorporations of MMA into the copolymer chains. The fitted curves serve to guide the eye. (d) Illustration of gradient compositional change following propagation orientation of copolymer grafting from silica nanoparticle surface using the RGB color coordinate (red, green, blue). For instance, 100 mol % PMMA represents (255, 0, 0), while 100 mol % PBA represents (0, 0, 255). By analogy, 50 mol % PMMA with 50% PBA represents (127, 0, 127).

The organic phase consisting of comonomers (including MMA and BA), surface-modified silica NPs (average core diameter *d* = 15.8 nm), and hexadecane (10.8 wt % compared to the comonomers) as a hydrophobe was placed on top of an aqueous phase (Scheme 1b). An anionic surfactant, sodium dodecyl sulfate (SDS), was added to facilitate the transportation of the Cu catalyst through interfacial and ion-pair catalysis, but an insufficient amount of Cu (<100 ppm vs monomers) would cause limited control over polymerization, as shown in a previous study.^55^ Therefore, to form the aqueous phase, 800 ppm catalyst complex [Br–Cu^II^(TPMA)]^+^ (TPMA = tris(2-pyridylmethyl)amine), SDS, and sodium bromide (NaBr) were dissolved in water. NaBr was added to suppress the dissociation of the Cu catalyst.^57^ The two phases were then homogenized, through 1 min of ultrasonication using a probe-sonicator, into a miniemulsion with a volume-averaged droplet hydrodynamic diameter of 116 nm (Figure S1). The small size of each droplet limited its capacity to accommodate on average around 11 silica NPs (Supporting Information, eq S2). Consequently, any cross-coupling occurring among the particle brushes during copolymerization should be effectively confined within these compartments instead of causing macroscopic gelation in the overall reaction mixture. Finally, ascorbic acid (AsAc), as a reducing agent, was slowly added (rate = 0.5 equiv h^–1^ vs [Cu^II^Br_2_(TPMA)]_0_ for 10 h) into the reaction mixture using a syringe pump to activate SI-ARGET ATRP. The optimal continuous feeding of AsAc was to ensure the most accessible initiating sites from the nanoparticle surface.^55^ Detailed reaction conditions are summarized in the caption of Figure 1, taking the initial comonomer feed molar ratio at 50:50 as an example. Upon full conversion, a small fraction of copolymerization products was considered to migrate out of the droplets. This could be caused by the cross-coupling of brush particles as the accommodation arrangement of other propagating analogues became more difficult.

Five different spontaneous gradient copolymers were grafted from surface-modified silica NPs (SiO_2_–Br). These particle brushes, denoted as SiO_2_M*x*B*y*, had varying initial feed molar ratios of MMA/BA of 70:30, 60:40, 50:50, 40:60, and 30:70. The copolymerization kinetics was monitored using ^1^H NMR and plotted in semilogarithmic coordinates (all detailed kinetics is shown in Figure S2). Three representative reactions for MMA/BA molar ratios of 70:30, 50:50, and 30:70 are shown in Figure 1a. For SiO_2_M7B3 with the highest MMA incorporation, both MMA and BA reached 96 and 95% conversions after 8 h, respectively. At 10 h, no observable vinyl proton peak was detected in the ^1^H NMR spectra, indicating complete conversion of both comonomers. We note that the semilogarithmic kinetic plots of SiO_2_M7B3 displayed a nonlinear trend. This could be attributed to a lower concentration of activators at the beginning (since AsAc was slowly injected). As the fraction of MMA decreased, slower polymerization rates were observed for both monomers. In the case of SiO_2_M3B7, when MMA showed 96% conversion after 24 h, BA had only 71% conversion and was not fully incorporated into the copolymer chains until 96 h (Figure S2i). Based on the conversions and initial concentrations of the comonomers, cumulative (eq 1) and instantaneous (eq 2) incorporations of MMA into the copolymer as a function of normalized chain length were plotted (Figure 1b,1c).^58^
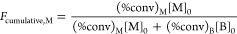
1
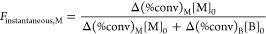
2

The initial PMMA contents (Table S1) could be predicted using the Mayo–Lewis eq (eq 3), where *r* and *f* represent the comonomer reactivity ratios and initial feed molar fractions, respectively.^59^
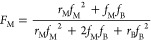
3

The cumulative and instantaneous additions of MMA into copolymers indicated a gradient compositional change along the chain propagation orientation. To visualize the gradient, the RGB (red, green, blue) three-color coordinate model was employed based on the fitted curve of compositional variance (Figure 1d). Interestingly, SiO_2_M7B3 with the highest MMA content showed the smallest variation in composition, with approximately 60 mol % PMMA at the final chain ends (most remote from the surface of the nanoparticle). A similar trend was also observed in SiO_2_M6B4, where approximately 30 mol % PMMA was present at the most “distal” (i.e., peripheral) brush layer positions. However, in the other three copolymer-grafted particle brushes (that initial MMA feed ≤50 mol %), there was almost no PMMA content at the chain ends, meaning that PBA-rich segments dominated at terminal positions of copolymer chains.

### Characterization of Dispersed and Gel-Fractions

**Figure 2:**
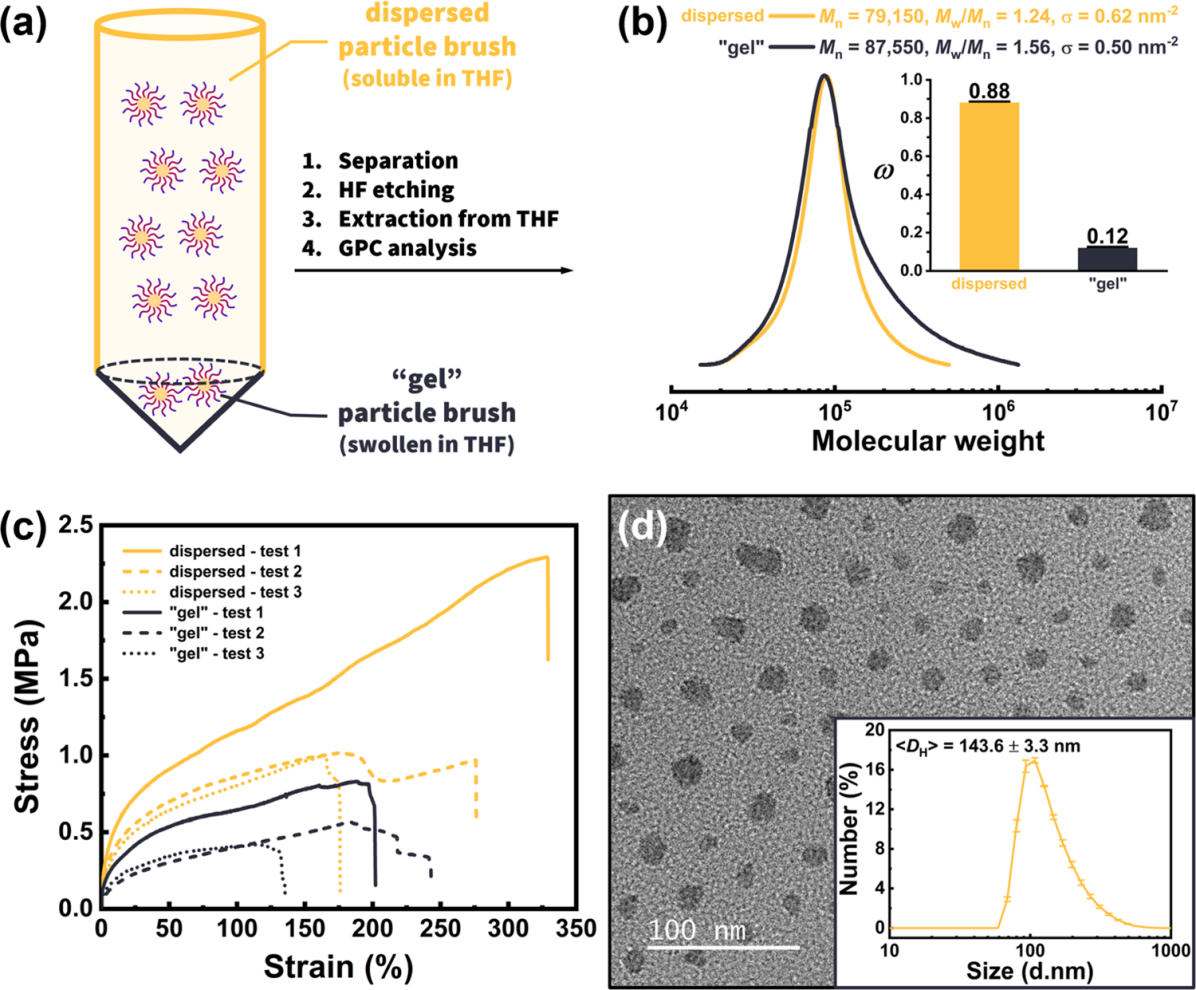
Characterization of spontaneous gradient copolymer-grafted particle brushes SiO_2_-*g*-PMMA-*grad*-PBA prepared by miniemulsion SI-ATRP (MMA/BA ratio of 50:50 mol %). (a) Illustration of dispersed and “gel” particle brushes that can be separated by centrifugation. While the dispersed particle brushes were fully soluble in THF as the supernatant, the “gel” particle brushes remained swollen and were centrifuged to the centrifuge tube bottom. After hydrofluoric acid (HF) etching, the dispersed and gel-fraction brush layers could be, respectively, extracted from THF. (b) GPC analysis of dispersed and “gel” brush layer; inset is the weight fractions of dispersed and “gel” products. (c) Strain–stress curves of the dispersed and “gel” samples (strain rate is 0.05 mm/s; testing temperature is 23 °C). (d) TEM image of the monolayer film of dispersed SiO_2_M5B5 after additional purification using ultrahigh speed centrifugation (16,000 × *g* for 1 h). Inset: number-averaged hydrodynamic diameter measured by DLS (∼10 mg/mL in THF).

After copolymerization, the particle brush products were initially purified by precipitation and centrifugation in methanol, followed by redissolution in tetrahydrofuran (THF) under high dilution. Most of the particle brushes could disperse well as a stable THF solution in the centrifuge tube. However, a small portion (referred to as “gel-fraction”) remained only swollen even after overnight sonication under high dilution (Figure 2a). To understand this phenomenon, another reaction using the same reaction conditions as those for SiO_2_M5B5 was repeated, and the resulting products were separated into dispersed and “gel-fraction” samples. Digital photos of dried dispersed samples showed translucent films adhering to the inner walls of glass vials. However, the dried “gel” samples formed disconnected small pieces, indicating sample inhomogeneity (Figure S3). The weight fraction of the residue (“gel”) part was approximately 12 wt % of all products.

It should be noted that while macroscopic gelation can be prevented in the heterogeneous reaction media, termination by combination of interparticle brush layers may still occur.^60^ Thermogravimetric analysis (TGA) indicated similar inorganic silica content from both the separated dispersed and “gel” brush nanoparticles (Figure S4). To further investigate the difference in molecular weight and distributions, gel permeation chromatography (GPC) analysis was conducted on the separated dispersed and “gel” brush, after etching the silica core with hydrofluoric acid (Figure 2b). The normalized refractive index signals indicated good control over polymerization, with a low dispersity (*M*_w_/*M*_n_ = 1.24) of the brush layer from dispersed samples. In contrast, the polymers extracted from the “gel-fraction” exhibited a broader but monomodal molecular weight distribution (*M*_w_/*M*_n_ = 1.56). The traces featured similar peak positions but showed a tailing into the high molecular weight and also a tailing into the lower molecular weight region, suggesting that the higher dispersity could originate from a slower deactivation (*k*_d_) in SI-ATRP equilibrium (where *k*_p_ is the rate of propagation) caused by the increased viscosity, at the high comonomer conversion stage (eq 4).^61,62^
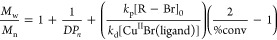
4

The interparticle termination could create a locally higher viscosity that could limit diffusion of the Cu^II^Br(Ligand) deactivator and slow down the deactivation process. For such a local gelation, it was enough to couple a few out of hundreds of chains grafted from each particle. The extent of termination was very small because no bimodality in GPC traces was observed.

A comparison of the mechanical properties between brush particle materials retrieved from the dispersed and gel-fraction was conducted using uniaxial stress–strain testing under room temperature (Figure 2c). Bulk films were fabricated by casting from THF solutions of dispersed samples and suspensions of “gel” counterparts. Tensile testing was performed following procedures outlined in a prior study.^52^ The Young’s modulus and toughness were derived from the initial slope and the integrated values of stress–strain curves, respectively (Figure S5). The results from three repeated tests for the dispersed samples yielded an average Young’s modulus of 18.0 ± 3.1 MPa, toughness of 2.9 ± 1.6 × 10^6^ J/m^3^, and ultimate strain of 259.3 ± 63.7%. These values surpassed those of the gel-fraction products (8.3 ± 3.1 MPa, 0.9 ± 0.3 × 10^6^ J/m^3^, and 182.7 ± 36.2%), indicating better mechanical properties of the dispersed particle brushes. This result demonstrates not only the relevance of microstructure uniformity to mechanical performance but also that structure uniformity needs to be considered for interpreting the mechanical properties of brush particle materials. Due to the limited processability, in the following, only samples pertaining to the dispersed fraction will be considered.

It was reported that ultrasonication, used to prepare and homogenize miniemulsions, could generate hydroxyl radicals from water.^63,64^ These radicals could initiate new linear polymer chains, not anchored on nanoparticle surfaces, leading to the presence of free polymer impurities.^55^ To minimize the amount of such impurities for subsequent mechanical testing, the dispersed products were subjected to ultrahigh speed centrifugation (16,000 × *g* for 1 h) for more efficient removal of the free copolymers. The centrifuged samples had low dispersities and high grafting density, indicating controlled copolymerizations (Table 1). The molar fractions of each component in SiO_2_M*x*B*y* determined by ^1^H NMR (Figure S6) provided further evidence of complete conversion copolymerizations of the particle brushes. Transmission electron microscopy (TEM) images of the monolayer film of purified particle brushes provided visual confirmation of the uniform distribution of SiO_2_ NPs in the copolymer matrices with minimal aggregation (Figures 2d and S7). Dynamic light scattering (DLS) measurement revealed a number-averaged hydrodynamic size of brush particles in THF of 144 nm. This agrees well with the estimated size of a brush particle in a good solvent given a degree of polymerization of 1110 (Supporting Information, eqs S3–S5).^65−68^ Moreover, the intensity-averaged distribution analysis (with an emphasis on larger brush nanoparticles) showed no evidence of aggregation of the particle brushes (Figure S8).

**Table 1:** Spontaneous Gradient Copolymer-Grafted Particle Brushes SiO_2_-*g*-PMMA-*grad*-PBA and Linear Analogues P(MMA-*grad*-BA) Prepared by Miniemulsion (SI-)ATRP

entry^a^	*x*, PMMA (mol %)^b^	*x*, PBA (mol %)^b^	*M*_n,app_ (×10^3^)^c^	*M*_w_/*M*_n_^c^	*f*_inorganic_ (%)^d^	σ (nm^–2^)^e^	*T*_g_ (°C)^f^	*T*_g_ range (°C)^f^
SiO_2_M7B3	70.8	29.2	83.5	1.32	7.3	0.53	52	[17, 74]
SiO_2_M6B4	61.0	39.0	77.3	1.25	7.5	0.56	33	[1, 67]
SiO_2_M5B5	50.3	49.7	81.7	1.26	6.1	0.66	5	[−31, 25]
SiO_2_M4B6	39.8	60.2	85.3	1.24	6.9	0.55	–9	[−49, 18]
SiO_2_M3B7	29.2	70.8	83.0	1.31	6.2	0.63	–20	[−55, 6]
LM7B3	71.8	28.2	73.2	1.29	N/A		50	[12, 66]
LM6B4	60.5	39.5	83.7	1.23			27	[5, 47]
LM5B5	50.7	49.3	85.5	1.24			11	[−15, 32]

a Reaction conditions are listed in Figure 1. SiO_2_M*x*B*y* and LM*x*B*y* represent the particle brushes initiated from the surface-modified silica nanoparticle surface (SiO_2_–Br) and linear copolymers initiated by a small-molecule initiator ethyl α-bromoisobutyrate (EBiB), respectively.

b Compositions of PMMA and PBA were determined by ^1^H NMR.

c Apparent molecular weight and distribution of copolymers were determined by THF GPC calibrated with PMMA standards.

d Inorganic fraction was determined by thermogravimetric analysis (TGA).

e Apparent grafting density (σ) was calculated from eq S1.

f Glass transition temperatures and ranges were determined by differential scanning calorimetry (DSC).

### Thermal and Mechanical Analyses

**Figure 3:**
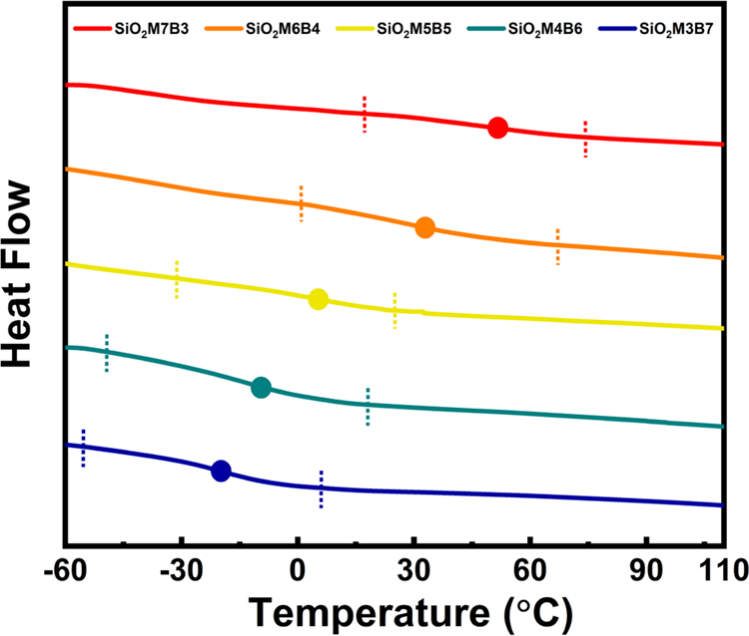
Thermal analysis by differential scanning calorimetry (DSC) on spontaneous gradient copolymer-grafted particle brushes SiO_2_-*g*-PMMA-*grad*-PBA. The dashed lines mark copolymer glass transition onsets and offsets, and the filled dot icons represent peak derivatives of the heat flow curves. Glass transition temperatures (*T*_g_) and ranges are summarized in Table 1.

Gradient copolymers are well-known for their unique physical properties, dependent on the nature of the compositional gradient. Examples are compositional heterogeneity, (potential) microphase separation, and exceptionally broad glass transition.^33,41^ The latter is located between the respective glass transition temperatures of each homopolymer component, resulting in the material possessing acoustic or vibrational damping capabilities by absorbing and dissipating energy within this broad transition region.^34,69^ To investigate the thermal behavior of the spontaneous gradient copolymer-grafted particle brushes, differential scanning calorimetry (DSC) was performed (Figure 3). The results demonstrated that all samples exhibited significantly broad glass transition regions (Table 1), with the minimum range of 56 °C (for SiO_2_M5B5) and the maximum range of 67 °C (for SiO_2_M4B6). Furthermore, a gradual shift in the transition ranges from the high-temperature region ([17, 74] for SiO_2_M7B3) to the low-temperature region ([−55, 6] for SiO_2_M3B7) was observed as the fraction of PMMA decreased. The specific experimental glass transition temperature values (*T*_g_s) were determined by analyzing the peak derivatives of the heat flow curves. These values were comparable to predictions obtained from the empirical Flory–Fox eq (eq 5, where ω is the weight fraction) based on the constituent fractions in the copolymers (Table S2).^70^
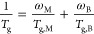
5

Among the copolymer brush particles, those with higher PBA fractions (SiO_2_M3B7 and SiO_2_M4B6) exhibited similar experimental and predicted *T*_g_s. However, the samples with higher PMMA fractions (SiO_2_M6B4 and SiO_2_M7B3) showed noticeable deviations. This deviation can be attributed to the *T*_g_ splitting due to the presence of minor PBA-rich and predominant PMMA-rich regions within the tethered copolymer brushes. This phenomenon has also been observed in a previous study on reverse-gradient copolymer brush particle materials.^52^ Therefore, when evaluating the overall copolymer composition and the shape of the gradient in samples with higher PMMA fractions, only the *T*_g_ values corresponding to the high-temperature region were considered.

**Figure 4:**
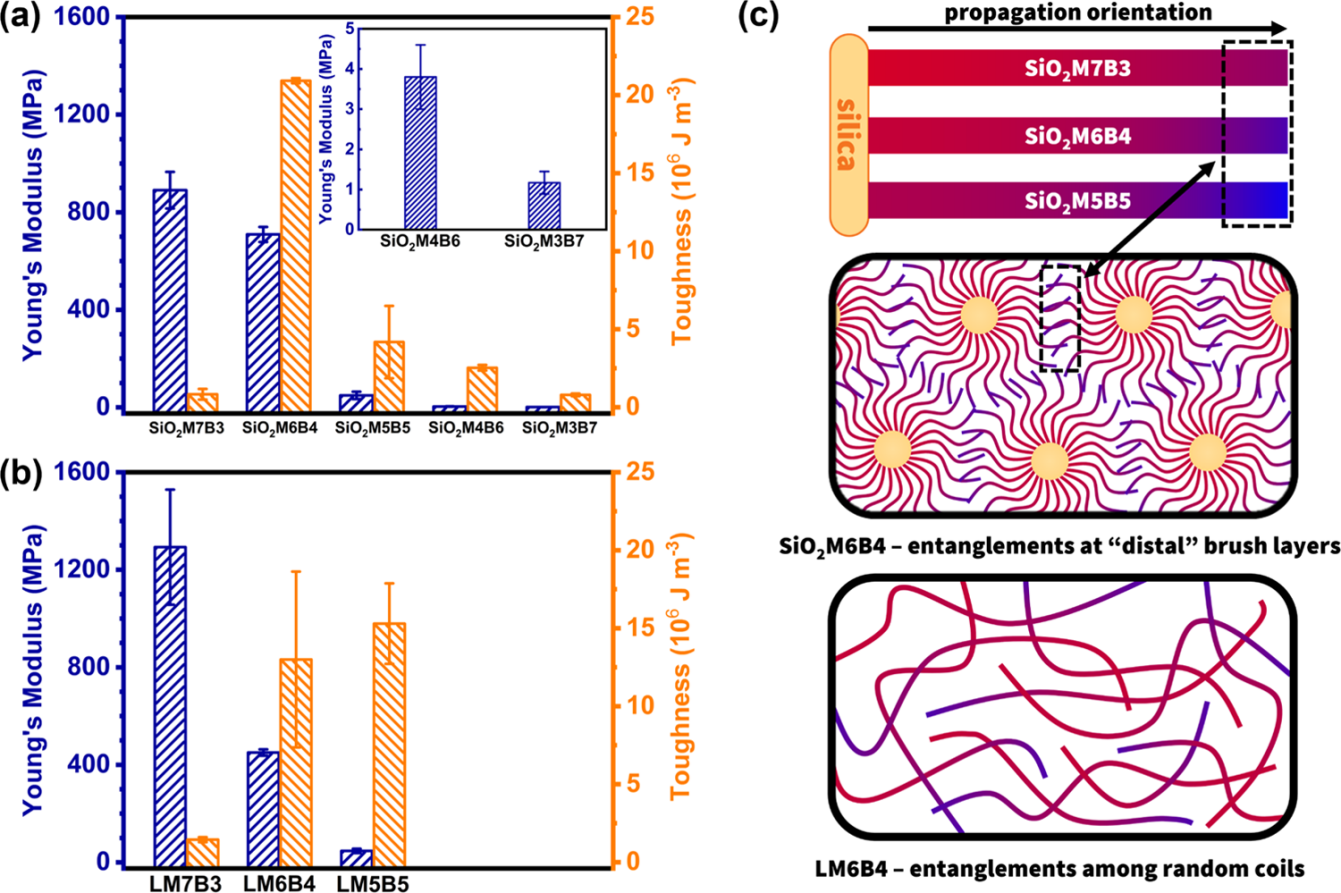
Mechanical analysis by uniaxial tension testing on spontaneous gradient copolymer-grafted particle brushes SiO_2_-*g*-PMMA-*grad*-PBA and linear analogues P(MMA-*grad*-BA) are used as a comparison. Determined Young’s modulus and toughness of (a) particle brushes SiO_2_-*g*-PMMA-*grad*-PBA and (b) linear P(MMA-*grad*-BA). (c) Illustration assumption on the synergistic mechanical property of SiO_2_M6B4. While material stiffness (herein modulus) is mainly determined by copolymer compositions, toughness is brought by interactions at “distal” grafted brush layers associated with the copolymer propagation orientations.

Apart from the broad glass transitions, gradient copolymers were also expected to show microstructural heterogeneity.^34,71,72^ This unique property was anticipated to provide the material with enhanced Young’s modulus and toughness compared with copolymers with a statistical sequence of similar compositions, evidenced by small-angle neutron scattering (SANS) referred to in our previous study.^47^ Mechanical analysis by uniaxial tension testing was conducted on the spontaneous gradient copolymer grafted from particle brushes under ambient conditions (23 °C) (Figure 4a). The results showed a clear decreasing trend in modulus with diminishing PMMA fractions in the copolymers, indicating that the material stiffness was primarily determined by the copolymer compositions. However, SiO_2_M6B4 had a notable maximal toughness of 20.9 ± 0.2 × 10^6^ J·m^–3^, much larger than in any other nanoparticle samples. This exceptional combination of high rigidity and ductility positioned SiO_2_M6B4 as a superior hybrid material.

Since it was not explicit why there was highest toughness for this specific copolymer composition of particle brushes, comparative testing was performed on spontaneous linear (unattached) gradient copolymers (Figure 4b). These linear samples, denoted as LM*x*B*y*, were synthesized using a small-molecule initiator ethyl α-bromoisobutyrate (EBiB) employing the aforementioned miniemulsion polymerization method (Table 1 and Figure S9). Only three copolymer compositions with higher PMMA fractions (MMA/BA ≥ 50:50 mol %) were prepared, as the others with lower glass transitions were expected to exhibit flow behavior at room temperature. To minimize testing deviations arising from macromolecular parameters, these linear analogues were designed with similar final copolymer compositions, molecular weights, and dispersities to their corresponding particle brushes.

The results illustrated that the material stiffness of the linear spontaneous gradient copolymers was consistently dominated by the copolymer compositions, with larger PMMA fractions resulting in materials with higher moduli. Although LM6B4 possessed considerable stiffness and toughness, as compared to SiO_2_M6B4, the expected peak value in toughness was absent and was slightly outperformed by LM5B5. Interestingly, LM5B5 exhibited significantly higher toughness than the corresponding particle brush sample, despite having a similarly low modulus. These results suggest that in the spontaneous gradient copolymers (both tethered and linear forms), the material stiffness is mainly determined by the copolymer compositions. Since toughness (defined as the area under a stress–strain curve) depends on the balance of stiffness and strain-to-fracture, we hypothesized the larger toughness of brush particle systems as compared to linear analogues could be caused by longer-range interactions between entanglement points (i.e., similar to ultrahigh molecular weight polymers) as well as a potential effect of the brush architecture on the distribution of repeat units and formation of heterogeneities (Figure 4c). As for particle brushes, the superior toughness of SiO_2_M6B4 can be attributed to the entanglement of terminal brush layers in a coiled semidilute polymer brush regime (SDPB),^73,74^ which consisted of approximately 35 mol % PMMA fractions. This would prevent the material from being excessively rigid (SiO_2_M7B3 with around 65 mol % PMMA) or soft (SiO_2_M5B5 with about 15 mol % PMMA) at the brush layer chain ends. Contrary to the conventional trade-off between material stiffness and toughness in linear polymers, the synergistic mechanical enhancement achieved in SiO_2_M6B4 by combining the composition and orientation exhibited the best overall material properties. This highlights the significance of the elaborate architectural design in shaping the gradient of brush particle materials.

## Conclusions

Grafting of spontaneous gradient poly(methyl methacrylate/*n*-butyl acrylate) copolymers with various compositional ratios from silica nanoparticles was successfully achieved at essentially complete conversion via miniemulsion SI-ARGET ATRP. Using heterogeneous reaction media effectively impeded interparticle brush coupling and prevented macroscopic gelation, even at high conversion. Gradient composition along the copolymer propagation orientation was determined by using ^1^H NMR. Upon precipitation in methanol and redispersion in THF, the dispersed particle brushes formed stable solutions, while the gel-fraction counterparts remained swollen even after sonication. They were separated through centrifugation. After etching the silica core, GPC analysis of the respective brush layers showed that the dispersed samples had narrow molecular weight distributions. At the same time, the “gel” products exhibited higher dispersity with a tailing into the high molecular weight region, attributed to slower deactivation in ATRP equilibrium under high viscosity conditions. Dispersed particle brushes showed better mechanical properties than the “gel” counterparts—monolayer film formed from the dispersed particle brushes displayed even nanoparticle distribution with minimal aggregation. DSC analysis revealed the characteristic broad glass transitions of spontaneous gradient brush layers, with glass transition temperatures increasing for higher PMMA compositions. Mechanical testing through uniaxial tension demonstrated that MMA/BA with “a golden ratio” of 60:40 in the copolymer brush layer exhibited both high stiffness and toughness. Comparison with the corresponding linear analogues of similar compositions highlighted the superior mechanical properties achieved through the synergistic effect of composition and orientation. The study emphasized the significance of architectural design in the tethered brush layers of hybrid materials, particularly in the “distal” brush region where interparticle brush interactions are predominant. Given the propensity for nanoparticle displacement only at high temperatures, we envision that these elaborately devised inorganic polymer hybrids hold the potential for self-repair upon heating, meanwhile maintaining high stiffness and toughness once cooled to room temperature as a potential self-healing material at “elevated” temperature.^49^

## Supporting Information Available

Experimental procedures; detailed kinetic studies of the spontaneous gradient copolymer brush layer, and additional characterization results of the dispersed and “gel” samples (PDF)
