# Supplementary material for: Composition-Orientation Induced Mechanical Synergy in Nanoparticle Brushes with Grafted Gradient Copolymers
Source: Macromolecules. 2023 Nov 29;56(23):9626–35. doi: 10.1021/acs.macromol.3c01799 (PMC10720466; doi:10.1021/acs.macromol.3c01799)
Supplement: Supplementary file 1 — ma3c01799_si_001.pdf [file ma3c01799_si_001.pdf]

Supporting Information for

Composition-Orientation Induced Mechanical Synergy in Nanoparticle Brushes with Grafted Gradient Copolymers

Rongguan Yin,[†] *Yuqi Zhao,*[‡] *Jaepil Jeong,*[†] *Jirameth Tarnsangpradit,*[‡] *Tong Liu,*[†] *So Young An,*[†] *Yue Zhai,*[‡] *Xiaolei Hu,*[†] *Michael R. Bockstaller,*[‡,*] *and Krzysztof Matyjaszewski*[†,*]

[†] Department of Chemistry, Carnegie Mellon University, 4400 Fifth Avenue, Pittsburgh, Pennsylvania 15213, United States.

[‡] Department of Materials Science and Engineering, Carnegie Mellon University, 5000 Forbes Avenue, Pittsburgh, Pennsylvania 15213, United States.

Contents

S1. Experimental Section. 2

S2. Characterization of Miniemulsion Droplets. 7

S3. Detailed Kinetic Studies of Complete Conversion Miniemulsion SI-ARGET ATRP. 9

S4. Characterizations of Dispersed and "Gel" Products. 12

S5. Glass Transition Temperatures. 20

References 22

* Corresponding author. Email: bockstaller@cmu.edu

* Corresponding author. Email: km3b@andrew.cmu.edu

S1. Experimental Section.

Materials. Silica nanoparticles (NPs), 30 wt% solution in methyl ethyl ketone (MEK-ST) were donated by Nissan Chemical and used as received. Tetherable initiator, 3-(chlorodimethylsilyl)propyl α-bromoisobutyrate (BiBSiCl), was synthesized as described in a previous report.[1] Sodium dodecyl sulfate (SDS, 99%, Aldrich), hexadecane (HD, 99%, Aldrich), sodium bromide (NaBr, 99%, Acros), ascorbic acid (AsAc, 99%, Aldrich), methanol (MeOH, > 99.8%, Aldrich), tetrahydrofuran (THF, 99.5%, Aldrich), toluene (99%, Aldrich), *N,N*-dimethylformamide (DMF, 99.9%, Acros), chlorodimethylsilane (98%, Aldrich), 48 wt % aqueous hydrofluoric acid (HF, 99.99%, Aldrich), allyl alcohol (99%, Aldrich), 2-bromoisobutyryl bromide (BiBB, 98%, Aldrich), sodium bicarbonate ($NaHCO_3$, ACS grade, Fisher Chemical), dichloromethane (DCM, 99%, Aldrich), sodium chloride (NaCl, ACS grade, Fisher Chemical), triethylamine (TEA, 99.5%, Aldrich), Karstedt's catalyst (platinum(0)-1,3-divinyl-1,1,3,3-tetramethyldisiloxane complex solution, in xylene, Pt ~2 %, Aldrich), ammonium hydroxide aqueous solution ($NH_3{\cdot}H_2O$, 28.0-30.0%, Fisher), alumina (neutral, Super I, 50-200μm, Sorbtech), anhydrous magnesium sulfate ($MgSO_4$, Fisher), and copper(II) bromide ($Cu^{II}Br_2$, 99.9%, Aldrich), Tris(2-pyridylmethyl)amine (TPMA, 99%, KOEI), tris(2-dimethylaminoethyl)amine (Me_6TREN, 99%, Alfa), tin(II) 2-ethylhexanoate ($Sn(EH)_2$, 95%, Aldrich), anisole (99%, Aldrich), were used as received unless otherwise stated. Stock solutions of $Cu^{II}Br_2$(TPMA) were prepared by dissolving $Cu^{II}Br_2$ and TPMA in a 1:1.1 molar ratio in water (0.05 M). *n*-Butyl acrylate (BA, Aldrich, > 99%), and methyl methacrylate (MMA, Acros, > 99%) were passed through a column filled with basic alumina prior to use to remove any polymerization inhibitor.

Analysis. Number-average molecular weight (M_n) and molecular-weight dispersity (M_w/M_n) were determined by gel permeation chromatography (GPC) equipped with Polymer Standards Services (PSS) columns (guard, 10^5, 10^3, and 10^2 Å) and a differential refractive index detector (Waters, 2410), with THF as eluent at a flow rate 1.00 mL/min (T = 35 °C). GPC traces were processed by WinGPC 8.0 software (PSS) using a calibration based on linear poly(methyl methacrylate) (PMMA) standards. All particle brush samples were etched with hydrofluoric acid (HF) for 12 h, neutralized with ammonia, and then processed through 1 mL neutral alumina column and a 450 nm PTFE filter before GPC measurement.[2] Comonomer conversion was determined by proton nuclear magnetic resonance (^{1}H-NMR) measurement. Particle size distributions were determined by using a Zetasizer Ultra (from Malvern Instruments Ltd) at 25 °C with water (for miniemulsion) or THF (for particle brushes, ~10 mg/mL) as dispersant. Samples were diluted prior to the measurement. Thermogravimetric analysis (TGA) was performed on a TA Instrument TGA 550 using air atmosphere, and the data was processed with TA Universal Analysis software. The heating procedure involved four steps: (1) ramp up at 20 °C/min to 120 °C; (2) hold at 120 °C for 10 min; (3) high-resolution ramp up at 20 °C/min to 800 °C; (4) hold at 800 °C for 5 min. The organic contents of the samples were normalized to the weight loss between 120 °C and 800 °C. The grafting densities were calculated using eq S1:

$$\sigma_{\mathrm{TGA}} = \frac{(1 - f_{\mathrm{SiO_2}}) N_A \, \rho_{\mathrm{SiO_2}} D_{\mathrm{core}}}{6 \, f_{\mathrm{SiO_2}} \, M_{\mathrm{n}}} \qquad \text{(eq S1)}$$

The value of $f_{\mathrm{SiO_2}}$ in the equation is the silica fraction measured by TGA after exclusion of any residual solvent; N_A is the Avogadro number; $\rho_{\mathrm{SiO_2}}$ is the density of silica NPs (2.2 g/cm^3); D_{core} is the average diameter of silica NPs (15.8 nm); and M_n is the number-average molecular weight of untethered polymeric brushes.

Transmission electron microscopy (TEM) was performed using a Philips Tecnai F20 electron microscope. All samples were dissolved in THF with concentration around 5 mg/mL, and solvent casting on carbon film supported Cu grids. To confirm results obtained from TEM, dynamic light scattering (DLS) described above with THF as dispersant was employed to determine number-averaged hydrodynamic diameter and intensity-weighted distribution.

Surface modification of silica nanoparticles. Silica nanoparticles were modified similarly as described in previous reports.[2, 3] Upon anchoring the tetherable initiators, 3-(chlorodimethylsilyl)propyl α-bromoisobutyrate (BiBSiCl), the surface-modified silica nanoparticles dissolved in methyl ethyl ketone underwent four cycles of dialysis (3 times methanol, 1 time acetone in dialysis bag with a 10 kDa cutoff) to remove the untethered initiators and other impurities. After dialysis, small scale SI-ATRP model reactions were completed separately at least three times to roughly calculate the accessible initiator density. Reaction conditions: SiO_2-Br 0.03g, MMA 3 mL, 50 vol% in anisole, $CuBr_2$ catalyst (in DMF, 0.005 g/mL) 200 ppm compared to monomer, [$CuBr_2$]:[Me_6TREN]:[Tin^{II}] = 1:3:5, 50 °C, 40 min reaction. The molecular weight of grafted brush layer was tested by THF GPC with PMMA standards after cleavage from silica using HF. The inorganic fractions of model particle brushes were determined by TGA. With the molecular weights and inorganic fractions, grafting densities were calculated by eq S1. The acquired average grafting density was assumed to be the accessible initiator densities (though the actual grafting densities may vary according to different monomer types). In this project, the molar concentration of tetherable initiator was determined to be 0.45 initiator molecules per nm^2 of silica nanoparticles (assuming a sphere with a diameter of 15.8 nm).

General procedure for the synthesis of spontaneous gradient poly(methyl methacrylate/*n*-butyl acrylate) copolymer grafted silica nanoparticle brushes (SiO_2-*g*-PMMA-*grad*-PBA) by full conversion miniemulsion SI-ARGET ATRP. Take MMA/BA molar ratio as 50/50 in sample SiO_2M5B5 for example. BA (2.41 g, 2.70 mL, 10.8 vol% in water), MMA (1.89 g, 2.00 mL, 8.0 vol% in water), silica nanoparticles (0.32 g, assuming ~0.45 surface Br/nm^2), and HD (0.43 g) were mixed to form the organic phase. $Cu^{II}Br_2$/TPMA stock solution 0.6 mL (0.05 M in 18.2 MΩ-cm ultrapure water), NaBr (0.26 g, 0.1 M), and SDS (0.27 g, 6.2 wt% to comonomers) were dissolved in 19.0 mL of ultrapure water. The organic and aqueous solutions were mixed (total volume ≈ 25.00 mL), placed in an ice bath, and homogenized by an ultrasonic probe sonicator, amplitude = 25 % for 1 min (application and rest time of 1 s each, 2 min in total). Nitrogen was bubbled into the miniemulsion for 20 min. The flask was immersed in a 50 ℃ oil bath and then AsAc solution (0.027 g/mL in ultrapure water) was slowly injected by a syringe pump. Mola ratios were as follows: $[MMA]_0/[BA]_0/[SiO_2\text{-Br}, \approx 0.45\ Br/nm^2]_0/[Cu^{II}Br_2(TPMA)]_0 = 500/500/1/0.8$. Samples were withdrawn periodically to follow the comonomer conversion by ^{1}H-NMR, while M_n and M_w/M_n of final products were determined by THF GPC (with PMMA standards). The final products were recovered by precipitation in methanol, redissolved in THF. The dispersed and "gel" particle brushes were separated by centrifugation (4800 rpm, 10 min). The dispersed samples as solutions in THF were kept, and further purified by ultrahigh speed centrifugation (Eppendorf 5418 Centrifuge, 16000×g for 1 h), dialysis in THF (with a 50kDa cutoff), and then dried in vacuum if necessary for further material characterizations.

Nuclear magnetic resonance spectroscopy (NMR). The comonomer conversions were analyzed by proton NMR (^{1}H NMR) using Bruker Avance™ III 500 MHz (^{1}H Frequency) NMR Instrument.

Differential scanning calorimetry (DSC) analysis. The glass transition temperatures (T_g) of copolymer grafted particle brushes were measured by DSC with TA Instrument QA-2000. The same procedure was repeated three times: (1) equilibrate at 25.00 °C; (2) isothermal for 1.00 min; (3) ramp 20.00 °C/min to -70.00 °C; (4) isothermal for 1.00 min; (5) ramp 20.00 °C/min to 160.00 °C; (6) isothermal for 1.00 min; (7) ramp 20.00 °C/min to -70.00 °C; (8) isothermal for 1.00 min; (9) ramp 20.00 °C/min to 160.00 °C; (10) isothermal for 1.00 min; (11) ramp 20.00 °C/min to -70.00 °C; (12) isothermal for 1.00 min; (13) ramp 20.00 °C/min to 160.00 °C; (14) isothermal for 1.00 min; (15) jump to 25.00 °C. The DSC data was analyzed with a TA Universal Analysis instrument, and T_g (range) was acquired.

Fabrication of a bulk film for mechanical analysis by uniaxial tension testing. Spontaneous gradient copolymer grafted particle brushes and linear spontaneous gradient copolymers were dispersed in THF as solution. Dispersions were transferred into 15 mm × 5 mm rectangular Teflon molds. The solvent was slowly evaporated over 48 h at room temperature generating transparent nanocomposite films with a thickness of 150-200 μm. The residual solvent was removed from the bulk films by transferring them to a vacuum oven at 120 °C for 24 h.

Tensile testing. The bulk films were tested in the tensile mode by using TA RSA-G2. The samples were stretched at a constant tensile rate of 0.05 mm/s at room temperature (approximately 23 °C).

S2. Characterization of Miniemulsion Droplets.

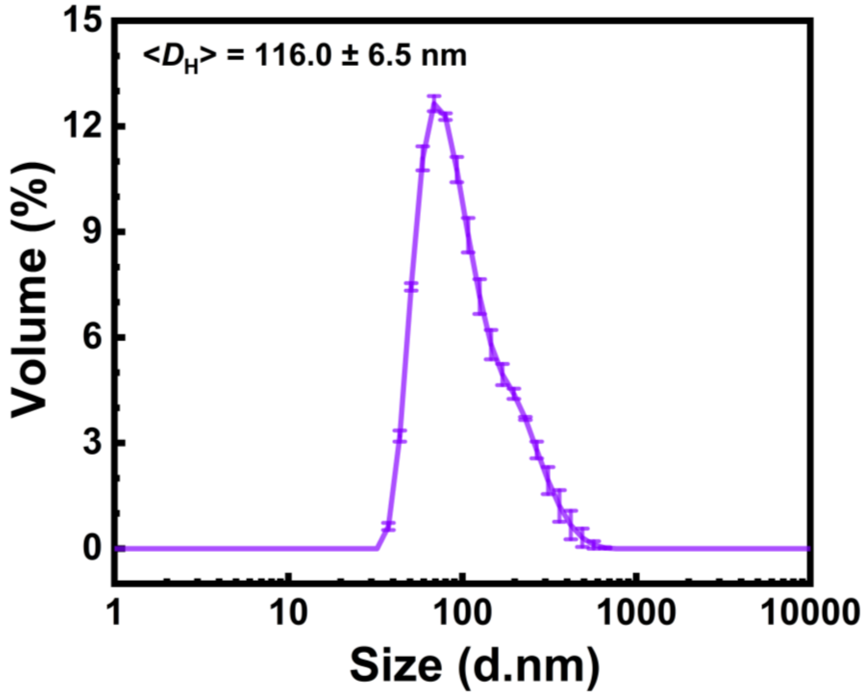

Figure S1. Volume-based average hydrodynamic size distribution by dynamic light scattering (DLS) measurements of typical miniemulsion droplets prepared by probe-sonicator for SI-ARGET ATRP.

The average number of silica NPs allocated inside each monomer droplet was estimated from:

$$N_{SiO_2/MD} = \frac{N_{\mathrm{SiO_2}}}{N_{\mathrm{MD}}} \qquad \text{(eq S2)}$$

- where the total number of silica NPs was calculated from: $N_{SiO_2} = \frac{m_{\mathrm{SiO_2}}}{\rho_{\mathrm{SiO_2}} V_{\mathrm{SiO_2}}}$, using $m_{\mathrm{SiO_2}}$ = 0.32 g, $\rho_{\mathrm{SiO_2}}$ = 2.2 g/cm^3 from reference,[2] and $V_{SiO_2} = \frac{4}{3}\pi r_{\mathrm{SiO_2}}^3$ ($r_{\mathrm{SiO_2}}$ = 7.9 nm).
- where the total number of miniemulsion droplets in dispersed media was calculated from: $N_{MD} = \frac{V_{organic}}{V_{MD}}$, using total volume of organic phase (MMA, BA, and hexadecane) V_{organic} = 5.26 cm^3 from Table 1, and $V_{MD} = \frac{4}{3}\pi r_{\mathrm{MD}}^3$ (average radius of droplets was estimated as r$_{MD}$ = 58 nm, according to the average hydrodynamic diameter measured by DLS).

Using these values, eq S2 provided $N_{SiO_2/MD} \approx 11$.

S3. Detailed Kinetic Studies of Complete Conversion Miniemulsion SI-ARGET ATRP.

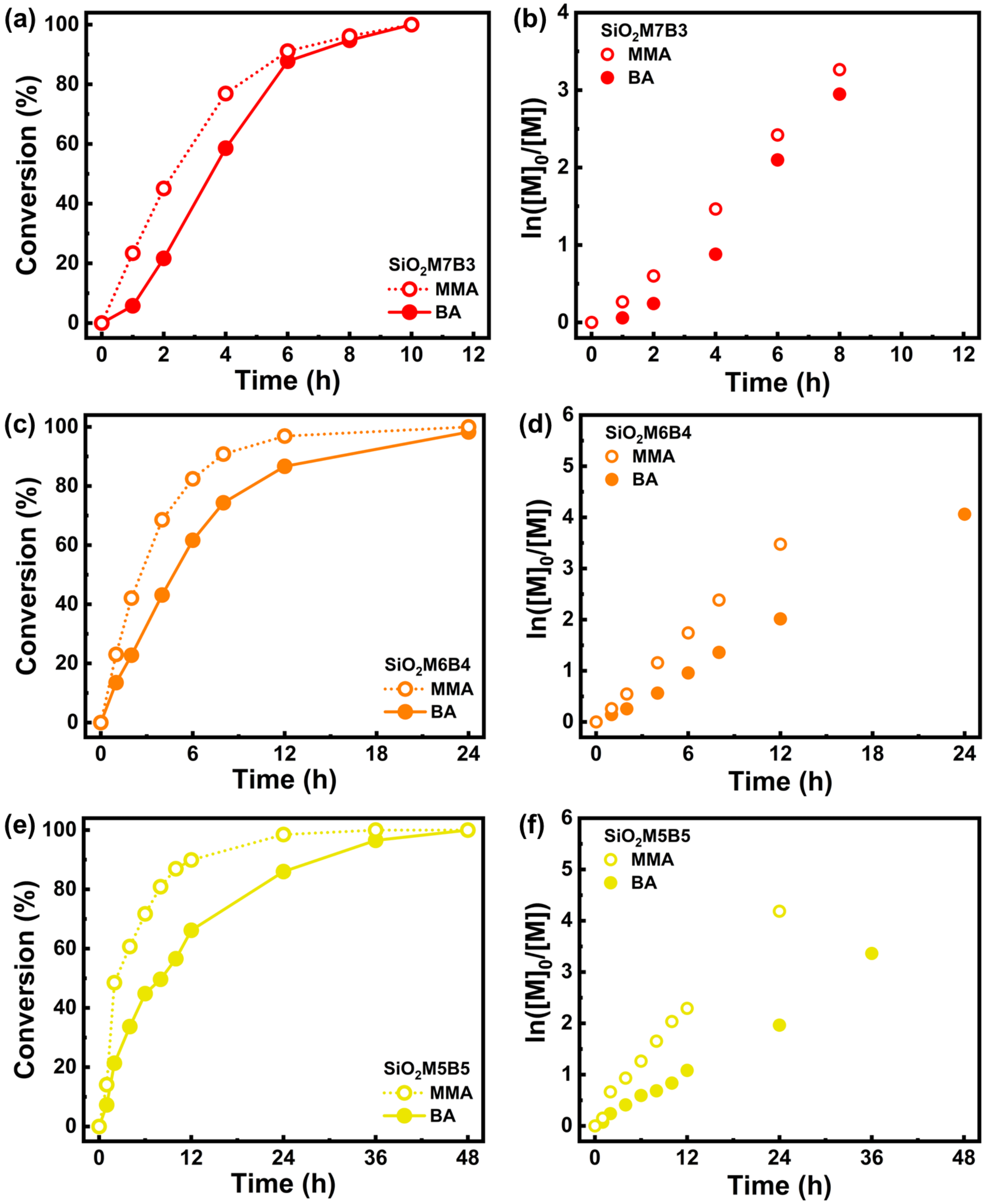

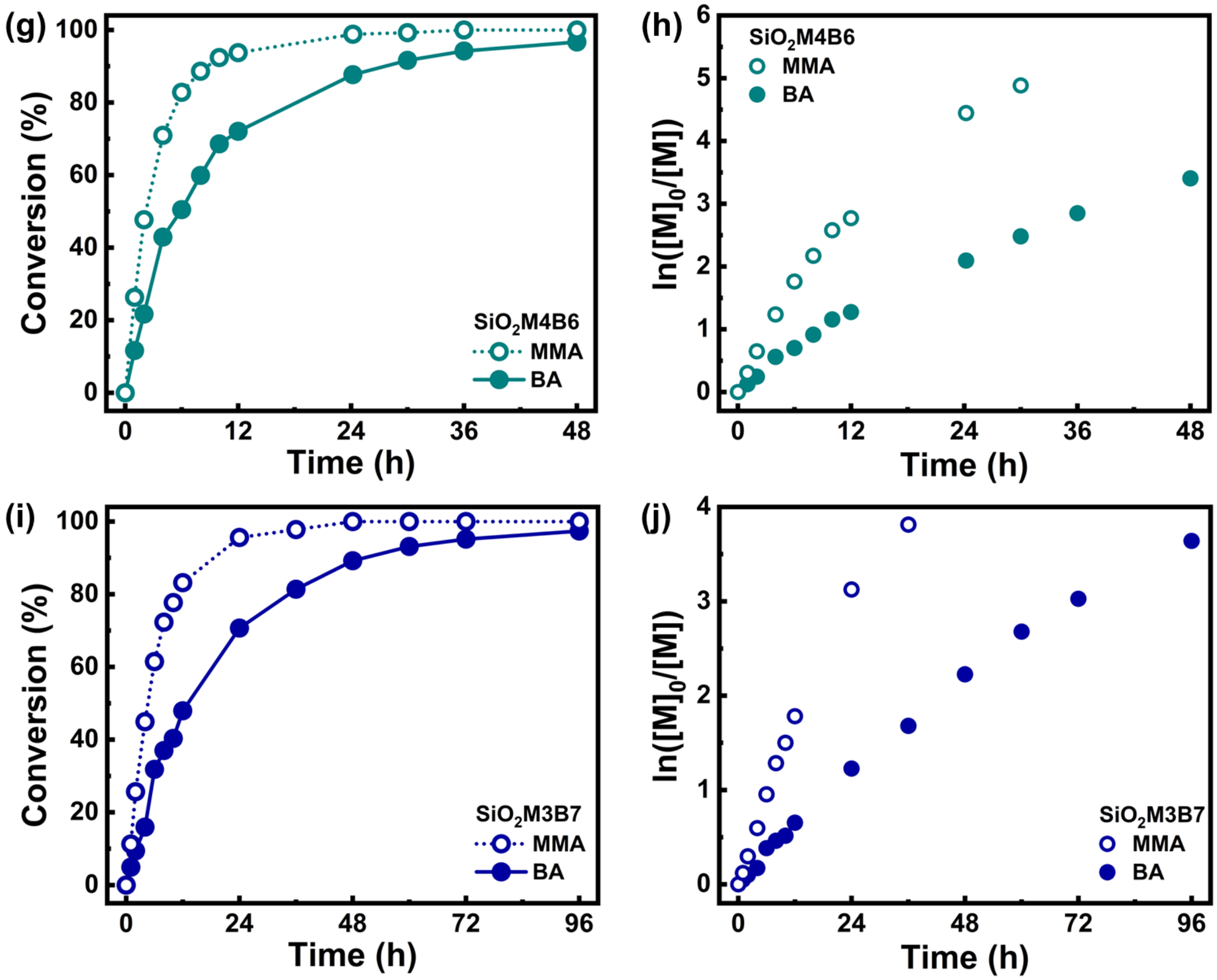

Figure S2. Comonomer conversions as a function of copolymerization time (a, c, e, g, i), and semilogarithmic kinetic plots (b, d, f, h, j) of spontaneous gradient poly(methyl methacrylate/*n*-butyl acrylate) copolymer grafted silica nanoparticle brushes (SiO_2-*g*-PMMA-*grad*-PBA) by full conversion miniemulsion SI-ATRP, with MMA/BA molar ratios of 70/30 (red, a and b), 60/40 (orange, c and d), 50/50 (yellow, e and f), 40/60 (green, g and h), and 30/70 (blue, i and j). Reaction conditions are listed as in Figure 1.

Table S1. Predicted Cumulative PMMA and PBA Contents at the Initiation Stage of the Copolymer Chains by Mayo–Lewis Equation (eq 3).

entry[a]	f_{MMA}[b]	f_{BA}[b]	F_{PMMA}[c]	F_{PBA}[c]
**SiO_2M7B3**	0.7	0.3	0.821	0.179
**SiO_2M6B4**	0.6	0.4	0.754	0.246
**SiO_2M5B5**	0.5	0.5	0.682	0.318
**SiO_2M4B6**	0.4	0.6	0.602	0.398
**SiO_2M3B7**	0.3	0.7	0.510	0.490

[a] The reactivity ratios for MMA and BA when copolymerizing: r_{MMA} = 1.79 and r_{BA} = 0.30, respectively.[4] [b] f_{MMA} and f_{BA} represent the initial comonomer feed molar fractions. [c] F_{PMMA} and F_{PBA} represent the initial PMMA and PBA contents predicted by the Mayo–Lewis equation (eq 3 in the manuscript), respectively.

S4. Characterizations of Dispersed and "Gel" Products.

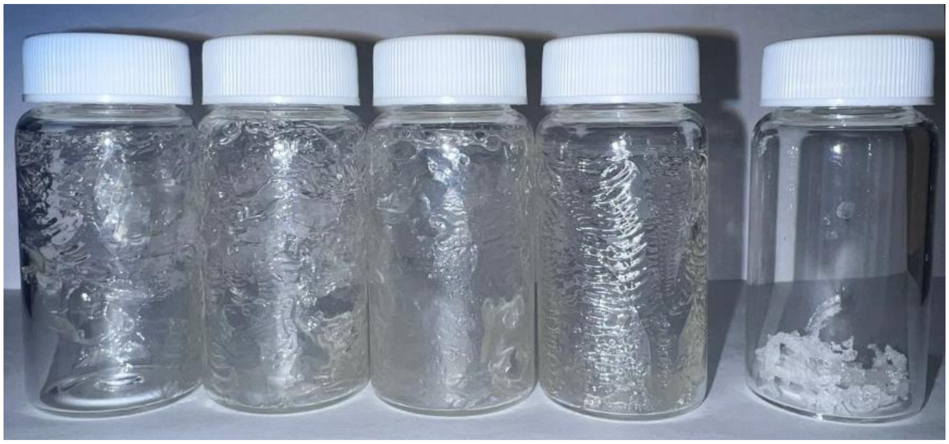

Figure S3. Digital photos of dried dispersed (the left 4 vials, m_{total} = 3.0830 g) and residue particle brushes (the right 1 vial, m_{total} = 0.4338 g). The total weight fractions of residue parts accounted for approximately 12.3 wt% among all products.

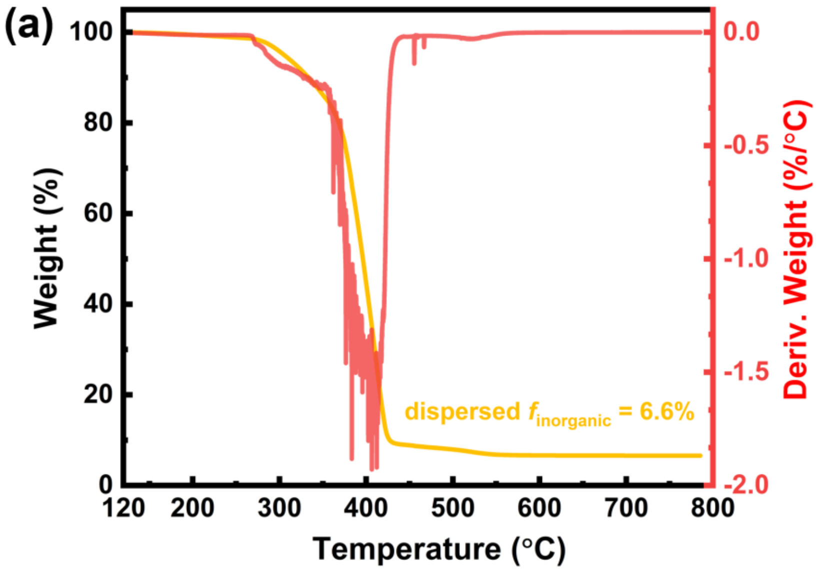

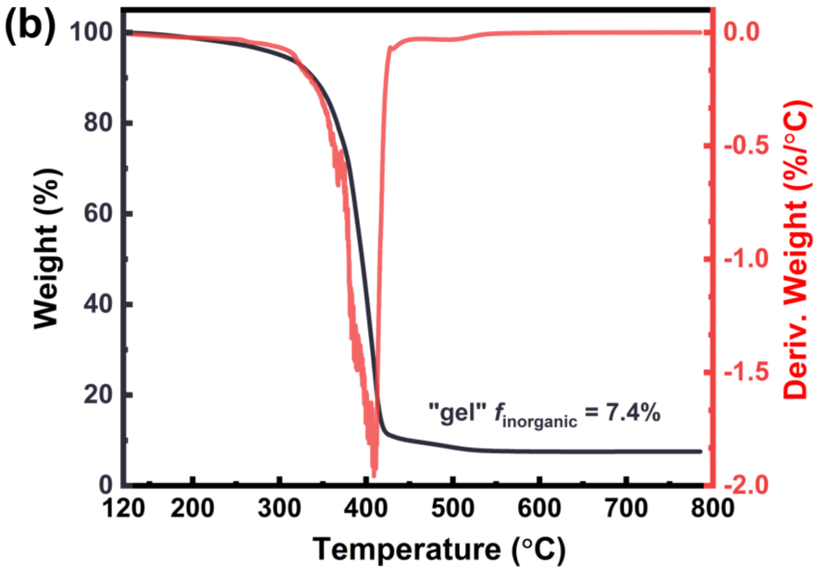

Figure S4. Thermogravimetric analysis (TGA) curves of dispersed and "gel" particle brushes showing the (derivative) weight loss as a function of temperature.

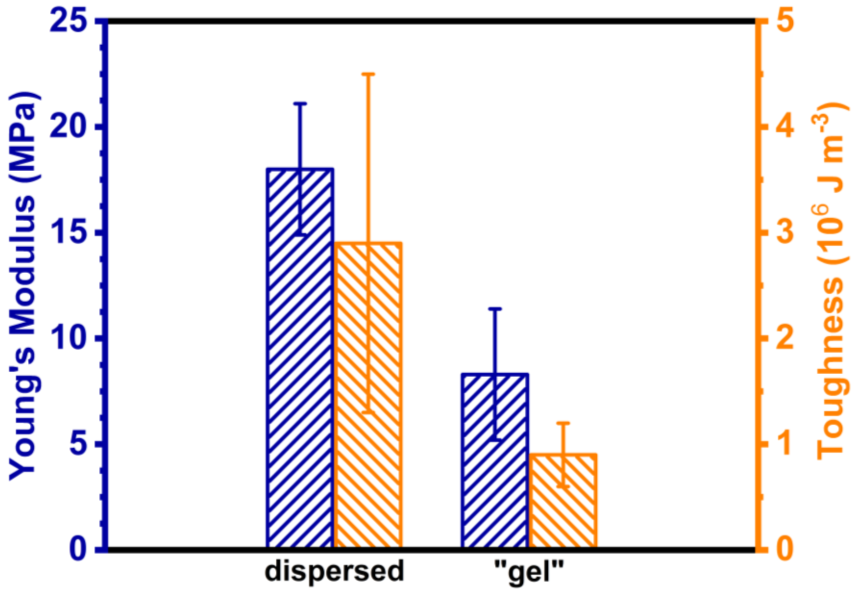

Figure S5. Young's modulus and toughness of dispersed and "gel" particle brush films determined by uniaxial tensile testing (strain rate 0.05 mm/s; testing temperature at 23°C). SiO_2-*g*-PMMA-*grad*-PBA samples were prepared by miniemulsion SI-ATRP (MMA/BA ratio of 50/50 mol%).

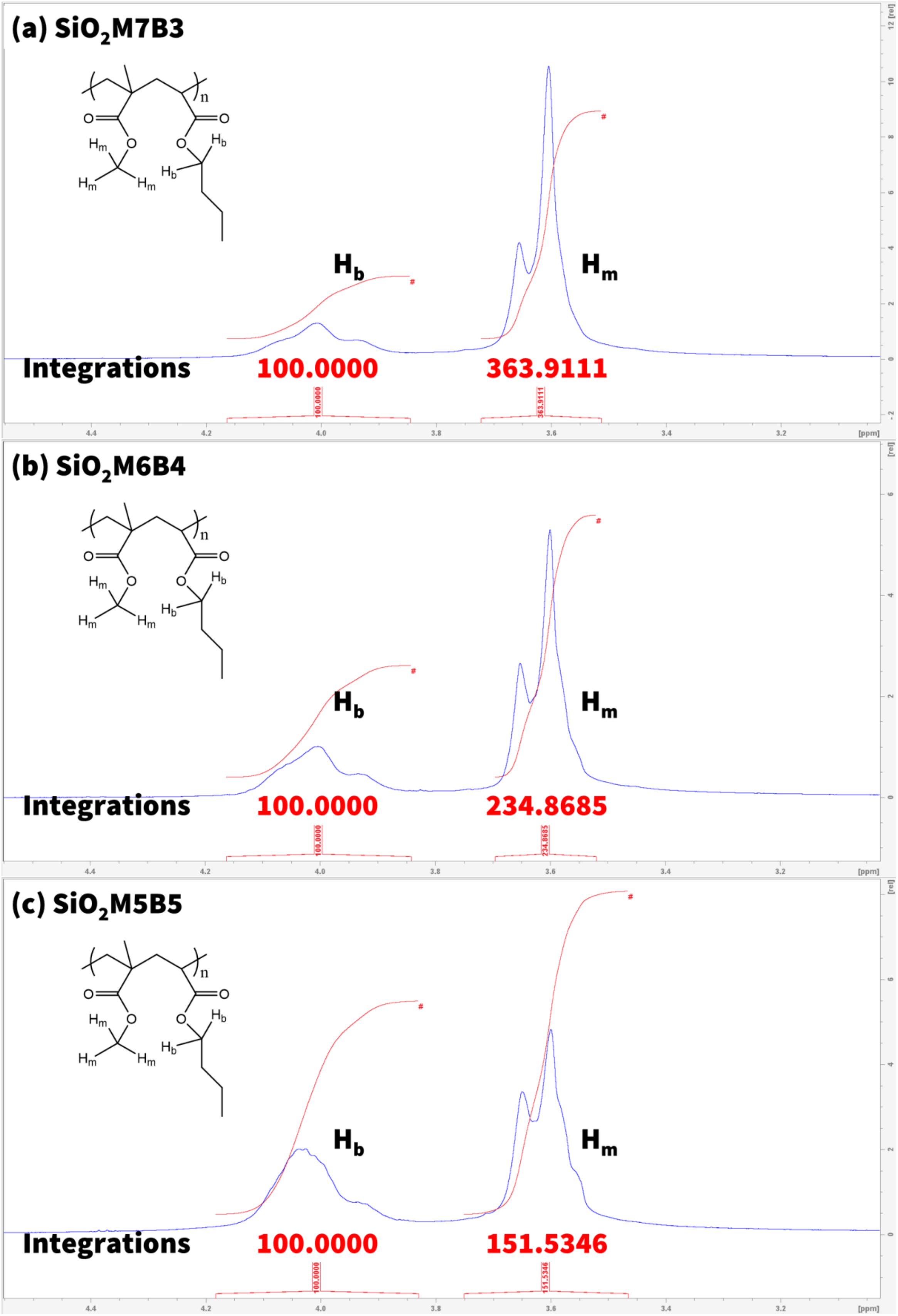
(a) SiO2M7B3
Hm
Hb
Integrations
100.0000
363.9111
(b) SiO2M6B4
Hm
Hb
Integrations
100.0000
234.8685
(c) SiO2M5B5
Hm
Hb
Integrations
100.0000
151.5346
[ppm]
[rel]

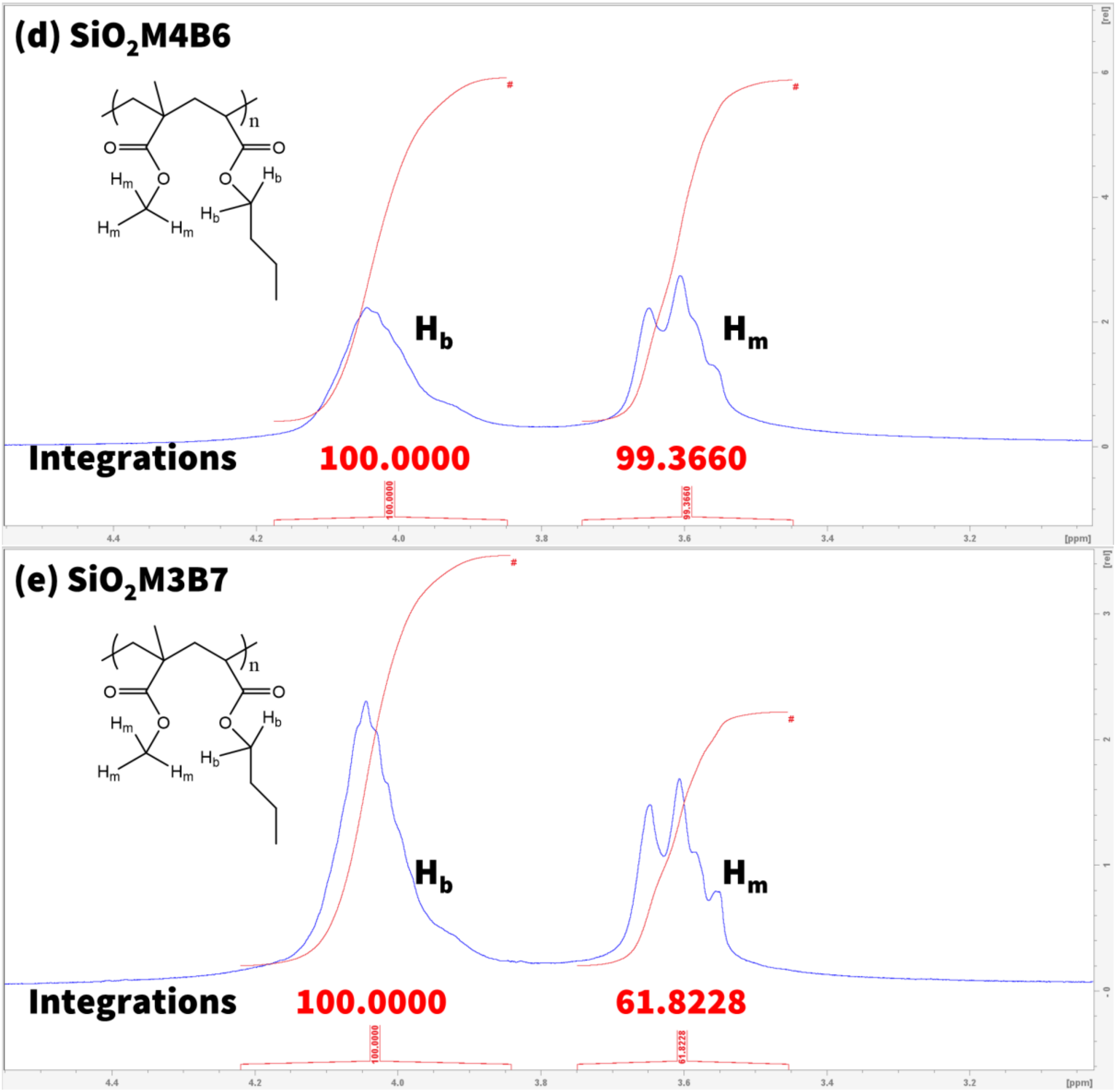

Figure S6. Proton nuclear magnetic resonance (^{1}H NMR) results of spontaneous gradient poly(methyl methacrylate/*n*-butyl acrylate) copolymer grafted silica nanoparticle brushes (SiO_2-*g*-PMMA-*grad*-PBA), with different PMMA/PBA molar fractions as (a) SiO_2M7B3, (b) SiO_2M6B4, (c) SiO_2M5B5, (d) SiO_2M4B6, and (e) SiO_2M3B7. The integrations of protons are near the ester group in BA (H_b) and MMA (H_m), respectively. Since there exist two H_b and three H_m, the molar ratios of PMMA to PBA taking SiO_2M7B3 as an example should be $\frac{x_{PMMA}}{x_{PBA}} = \frac{363.9111 \div 3}{100.0000 \div 2} \approx \frac{0.708}{0.292}$.

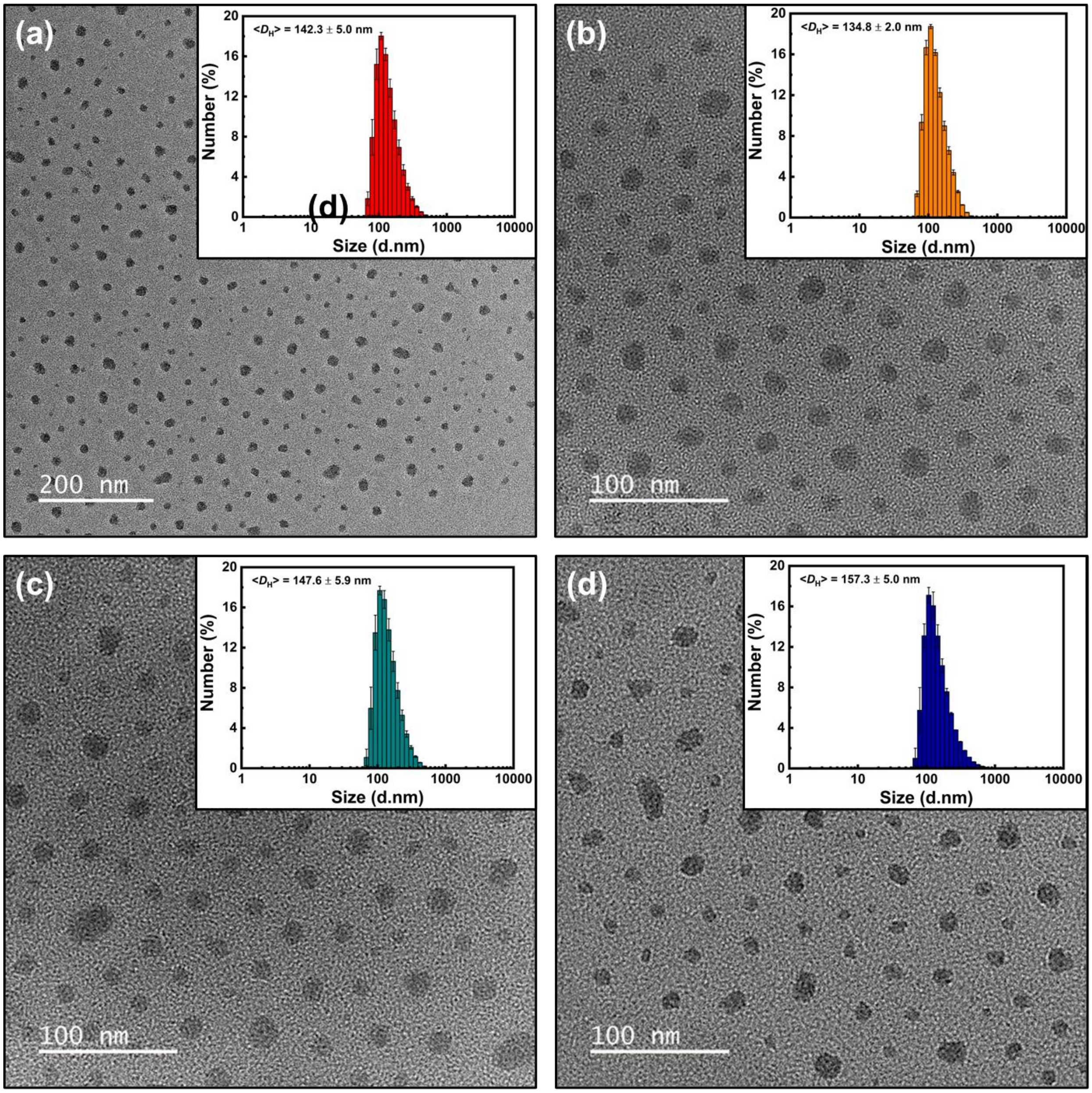

Figure S7. TEM images of monolayer film of dispersed particle brushes (a) SiO_2M7B3, (b) SiO_2M6B4, (c) SiO_2M4B6, and (d) SiO_2M3B7 after additional purification using ultrahigh speed centrifugation (16000×g for 1 h). Inset: number-averaged hydrodynamic diameter measured by DLS (~10 mg/mL in THF).

Degree of Polymerization Approximation of Particle Brush

Assume scaling of 0.98 and 0.58 based on "concentrated particle brush" (CPB) and "semidilute particle brush" (SDPB), respectively.[5]

The characteristics of our particle brush (PB) include a core diameter (d_0) of ~ 15 nm, particle size based on DLS (d_{DLS}) of 144 nm, and the surface grafting density (σ_0) of 0.66 chain/nm^2. To distinguish the CPB and SDPB regime, we calculate the critical radius (r_c) where the transition from CPB to SDPB occur based on Fukuda-Ohno model as follows,[6]

$$r_c = r_0 \sigma_0^{*1/2} \nu^{*-1} \qquad \text{(eq S3)}$$

Here, σ_0^* is the reduced grafting density which can be determined from $\sigma_0 a^2$, and ν^* is the reduced excluded volume parameter which is proportional to the excluded volume parameter $= \nu/\sqrt{4\pi}$ (detailed derivation can be found in reference).[6] Based on eq S3, the calculated r_c of our PB is 35.1 nm. This means that r_c is larger than the core radius ($r_0 = d_0/2$) and is smaller than the particle radius ($r = d_{DLS}/2$). Therefore, PB consists of both CPB and SDPB regimes.

To determine the degree of polymerization (*N*), this can be derived from the known size of the polymer shell, $h = r - r_0$. Applying the previously obtained scaling from DLS of 0.98 and 0.58 in CPB and SDPB, respectively, gives:

$$r - r_0 = aN_c^{0.98} + a(N - N_c)^{0.58} \qquad \text{(eq S4)}$$

where the first term on the right-hand side corresponds to CPB and the second term to the SDPB. N_c is the critical degree of polymerization corresponding to r_c whereas a is the segmental length of the polymer shell. Since the length from particle core surface to CPB-SDPB transition can be determined from the known CPB scaling (i.e., $r_c - r_0 = aN_c^{0.98}$), we can rewrite eq S4 to then obtain the following:

$$N = \left(\frac{r_c - r_0}{a}\right)^{\frac{1}{0.98}} + \left(\frac{r - r_c}{a}\right)^{\frac{1}{0.58}} \qquad \text{(eq S5)}$$

a is approximated as 0.65 nm based on the known statistical segment length of PMMA.[7, 8] With these known parameters, we can calculate N to be 1110.

(Note) Since the actual particle brush grafts P(MMA-*grad*-BA) copolymer, this part is a rough approximation (based on PMMA homopolymer).

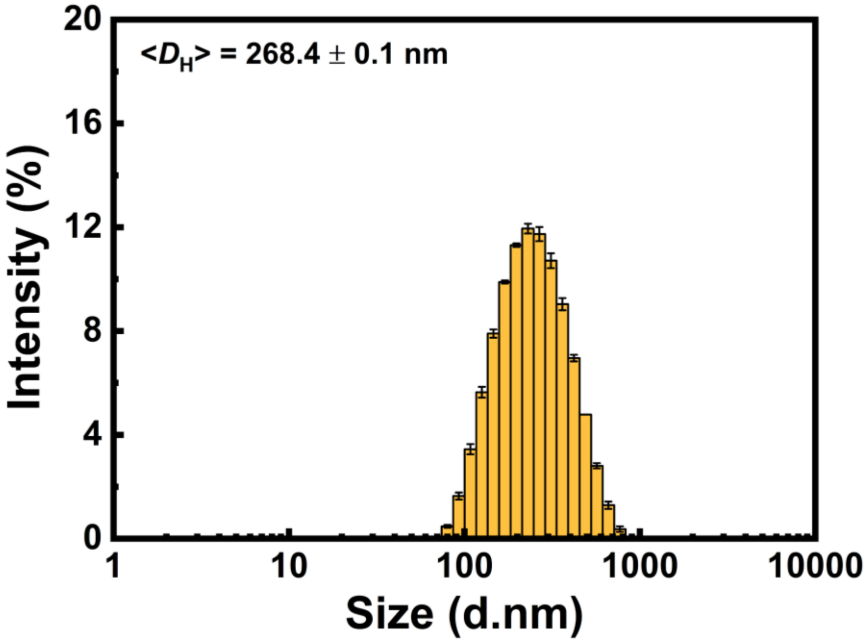

Figure S8. Intensity-averaged hydrodynamic diameter measured by DLS (~10 mg/mL in THF) of dispersed SiO_2M5B5 samples after additional purification using ultrahigh speed centrifugation (16000×*g* for 1 h).

S5. Glass Transition Temperatures.

Table S2. Predicted Glass Transition Temperatures (T_g) of Spontaneous Gradient P(MMA-*grad*-BA) Copolymer Calculated by the Empirical Flory–Fox Equation (eq 5).

entry	x, PMMA (mol%)[a]	x, PBA (mol%)[a]	ω_M[b]	ω_B[b]	T_g (K)	T_g (°C)
**SiO_2M7B3**	70.8	29.2	0.654	0.346	303.2	30.0
**SiO_2M6B4**	61.0	39.0	0.550	0.450	288.5	15.4
**SiO_2M5B5**	50.3	49.7	0.442	0.558	274.8	1.6
**SiO_2M4B6**	39.8	60.2	0.341	0.659	263.1	-10.0
**SiO_2M3B7**	29.2	70.8	0.244	0.756	252.8	-20.3

[a] The overall PMMA and PBA fractions in the copolymers were measured by ^{1}H NMR (Table 1 and Figure S6). [b] Weight fractions were calculated based on molar mass of MMA = 100.12 g·mol^{-1}, and BA = 128.17 g·mol^{-1}. The glass transition temperatures of PMMA homopolymer (M_n = 37,300, $Đ$ = 1.25) and PBA homopolymer (M_n = 38200, $Đ$ = 1.49) measured by DSC were 91 ($T_{g,M}$) and -43 ℃ ($T_{g,B}$), respectively.[9]

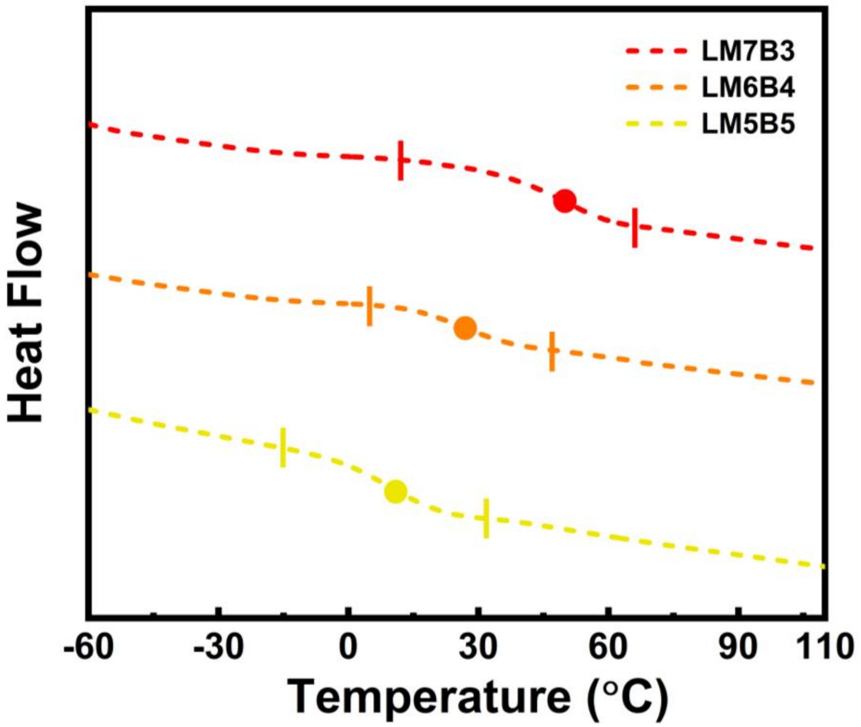

Figure S9. Thermal analysis by differential scanning calorimetry (DSC) on spontaneous gradient linear copolymer analogues P(MMA-*grad*-BA). The dash lines mark copolymer glass transition onsets and offsets, and the filled dot icons represent peak derivatives of the heat flow curves. Glass transition temperatures (T_g) and ranges are summarized in Table 1.

References

1. Yan, J.; Pan, X.; Wang, Z.; Zhang, J.; Matyjaszewski, K., Influence of Spacers in Tetherable Initiators on Surface-Initiated Atom Transfer Radical Polymerization (SI-ATRP). *Macromolecules* **2016,** *49* (23), 9283-9286.
2. Yan, J.; Kristufek, T.; Schmitt, M.; Wang, Z.; Xie, G.; Dang, A.; Hui, C. M.; Pietrasik, J.; Bockstaller, M. R.; Matyjaszewski, K., Matrix-free Particle Brush System with Bimodal Molecular Weight Distribution Prepared by SI-ATRP. *Macromolecules* **2015,** *48* (22), 8208-8218.
3. Yan, J.; Pan, X.; Schmitt, M.; Wang, Z.; Bockstaller, M. R.; Matyjaszewski, K., Enhancing Initiation Efficiency in Metal-Free Surface-Initiated Atom Transfer Radical Polymerization (SI-ATRP). *ACS Macro Lett.* **2016,** *5* (6), 661-665.
4. Dubé, M. A.; Penlidis, A., A systematic approach to the study of multicomponent polymerization kinetics—the butyl acrylate/methyl methacrylate/vinyl acetate example: 1. Bulk copolymerization. *Polymer* **1995,** *36* (3), 587-598.
5. Choi, J.; Hui, C. M.; Pietrasik, J.; Dong, H.; Matyjaszewski, K.; Bockstaller, M. R., Toughening fragile matter: mechanical properties of particle solids assembled from polymer-grafted hybrid particles synthesized by ATRP. *Soft Matter* **2012,** *8* (15), 4072-4082.
6. Ohno, K.; Morinaga, T.; Takeno, S.; Tsujii, Y.; Fukuda, T., Suspensions of Silica Particles Grafted with Concentrated Polymer Brush: Effects of Graft Chain Length on Brush Layer Thickness and Colloidal Crystallization. *Macromolecules* **2007,** *40* (25), 9143-9150.
7. Haley, J. C.; Lodge, T. P., Dynamics of a poly(ethylene oxide) tracer in a poly(methyl methacrylate) matrix: Remarkable decoupling of local and global motions. *J. Chem. Phys.* **2005,** *122* (23), 234914.

8. Zeroni, I.; Lodge, T. P., Chain Dimensions in Poly(ethylene oxide)/Poly(methyl methacrylate) Blends. *Macromolecules* **2008,** *41* (3), 1050-1052.

9. Yin, R.; Zhao, Y.; Gorczyński, A.; Szczepaniak, G.; Sun, M.; Fu, L.; Kim, K.; Wu, H.; Bockstaller, M. R.; Matyjaszewski, K., Alternating Methyl Methacrylate/*n*-Butyl Acrylate Copolymer Prepared by Atom Transfer Radical Polymerization. *ACS Macro Lett.* **2022,** *11* (10), 1217-1223.
